# Knowledge and information sources towards *Helicobacter pylori* in Jordan

**DOI:** 10.1371/journal.pone.0278078

**Published:** 2023-03-08

**Authors:** Nader Alaridah, Raba’a F. Jarrar, Rayan M. Joudeh, Mallak Aljarawen, Mohammad Jum’ah, Hasan Nassr, Raad Riad AlHmoud, Abdullah Allouzi, Eslam M. Wadi, Anas H. A. Abu-Humaidan

**Affiliations:** 1 Department of Pathology, Microbiology, and Forensic Medicine, School of Medicine, The University of Jordan, Amman, Jordan; 2 Department of the Clinical Laboratory Sciences, School of Science, The University of Jordan, Amman, Jordan; 3 College of Medicine, Sulaiman Al-Rajhi University, Al-Bukayriah, Al-Qassim, Saudi Arabia; 4 School of Medicine, The University of Jordan, Amman, Jordan; 5 Faculty of Medicine, Al-Balqa Applied University, As-Salt, Jordan; 6 Faculty of Medicine, Mu’tah University, Karak Governate, Jordan; University of Petra (UOP), JORDAN

## Abstract

In 2017, the Jordanian Ministry of Health reported that gastric cancer was one of Jordan’s most diagnosed cancers. Gastric cancer is often linked to *Helicobacter pylori*, one of the foremost risk factors. Despite the high prevalence of *H*. *pylori* in Jordan, no information is available regarding the general population’s awareness of the harmful effects of this pathogen. The study aims to assess the knowledge and the impact of the source of knowledge on *H*. *pylori* among the general population in Jordan. A cross-sectional study involving 933 participants was conducted between May and July of 2021. After meeting the inclusion criteria and consenting to participate in this study, participants completed the questionnaire. An interview-based questionnaire covered the following sections: sociodemographic data and knowledge related to *H*. *pylori* infection. 63% of the participants had a high level of education, 70.5% got their information about *H*. *pylori* infection from non-medical sources, and 68.7% had a low level of knowledge. Working in the medical field, attaining information from medical resources, and having a history of self-reported or family member of *H*. *pylori* infection showed a significant association with a high level of knowledge. The Mann-Whitney U test showed that the mean ranks of all knowledge items in the medical source group significantly exceed those of the non-medical source group (p-values < 0.05). In Jordan, the awareness of *H*. *pylori* was unsatisfying, like in other countries. Nevertheless, misconceptions in knowledge about *H*. *pylori* were identified and further awareness must be spread and advocated. Close observation of the non-medical sources of information is essential for delivering sufficient amount of knowledge to the general population.

## Introduction

*Helicobacter pylori*, a bacterium spotlighted the interest of many researchers to dig deep about this organism which become a global concern to eradicate it and diminish the complications arising such as gastric cancer [1]. Gastric cancer is ranked as the fifth most common cancer worldwide with one million new cases associated with *H*. *pylori* infection by 2020 [2]. Eastern Asia had the highest rates of gastric cancer worldwide for both genders [3]. Unfortunately, the Hashemite Kingdom of Jordan has no updated statistical or epidemiological findings about gastric cancer. In 2017, the Jordanian Ministry of Health reported that gastric cancer has ranked ninth among the most diagnosed cancers with 3.3% of the total cancer cases in Jordan [4]. Moreover, gastric cancer is considered the seventh and tenth most frequent cancer among Jordanian males and females, respectively. Statistical information about gastric cancer in Jordan is deficient due to the lack of any national screening system. In addition, such high prevalence is similarly displayed in other developing countries around the world [5, 6]. Interestingly, one cross-sectional study found that approximately 88% of the Jordanian population tested positive for *H*. *pylori* [7]. On the other hand, developed countries have shown a lower prevalence than developing countries [8]. This can be linked and associated with various poor hygienic routines of facilities and infrastructural bases [9]. Knowledge is one of the most critical factors associated with the high prevalence of *H*. *pylori* among the Jordanian population. Globally, knowledge and awareness are inadequate regarding *H*. *pylori* infection among the general population [10]. Lamentably and to our knowledge, there is no published data about the level of knowledge of the Jordanian general population for *H*. *pylori* infection. The value of the knowledge acquired is influenced greatly by sources of information used. There are various sources of information to obtain related health information including doctors, healthcare professionals, TV, radios, books, internet, and social media networks. A study conducted in Jordan showed that specialists were the most used source of information among the general population [11]. On the other hand, one cross-sectional study done in Saudi Arabia showed that participants are immensely influenced by the information provided through social media platforms [12]. Such findings [11, 12] indicate the need to assess the validity, accuracy, and adequacy of different information sources about the knowledge of general population. The primary aim of this study is to assess the level of knowledge and investigate the sociodemographic predictors with high level of knowledge. The secondary aim was to identify numerous scopes of information sources that will influence the level of knowledge about *H*. *pylori* among the Jordanian general population as well as the areas of improvement needed to reach the optimum understanding and sufficient knowledge about *H*. *pylori* infection.

## Materials and methods

### Sample recruitment and study setting

A Descriptive cross-sectional study was carried out at Jordan University Hospital between May and July of 2021. Outpatient Visitors of Jordan University Hospital were encouraged to voluntarily take part in the study. Participants had to be at least 18 years old, able to converse verbally in Arabic, and willing to engage in the study to be enrolled. Those who did not match the inclusion criteria were excluded. Convenience sampling method was used to recruit the participants from outpatient clinic visitors in different clinics. Medical students from 5^th^ and 6^th^ years were trained by the research team to interview the volunteered visitors. Participants who agreed to participate in this study answered interview-based questions about their *H*. *pylori* knowledge and the source of the information used. The questionnaire took 8 minutes to complete. The estimated minimum sample size needed for the study is 385, this was calculated based on a 5% margin of error and 50% prevalence [13].

### Development of the survey

A questionnaire has been designed based on a literature review [14] and other studies [15–17]. As there is no validated tool available to assess community knowledge. The questionnaire was written in Arabic. After the survey was finished, it was analyzed and verified by gastroenterologists and microbiologists. Following pilot testing, certain linguistic and grammatical changes were made to ensure that the questions were comprehensible. The internal consistency was evaluated, and Cronbach’s alpha of knowledge was calculated (0.83).

### Measurement tool

Data were gathered using an Arabic-based online questionnaire composed of the following sections: participants’ background characteristics and general knowledge. Sociodemographic characteristics were obtained including age, gender (male, female), marital status (married, unmarried), educational level (high education, low education), occupational status (medical field, non-medical field, not working), self-reported history of *H*. *pylori* infection (yes, no), family history of *H*. *pylori* (yes, no), current residence (urban/rural housing), and source of information acquired about *H*. *pylori* (medical source [i.e. directly from doctors, nurses, pharmacists, etc., or educational field], non-medical sources [i.e. from a family/ friends or social media or TV/Radio, etc.]).

The questionnaire had 43 questions about different topics regarding *H*. *pylori*. The questions are divided into seven main items to assess the level of knowledge about *H*. *pylori*, the items are: 1) nature of *H*. *pylori*. [i.e., is it virus, bacteria, parasite or worm?] 2) organs affected by *H*. *pylori*, 3) mode of transmission, 4) common signs and symptoms, 5) methods of diagnosis, 6) type of treatment, 7) general knowledge about *H*. *pylori* including prevalence in Jordan, important risk factors, and complications. The answers to the questions were as the following: (Yes, no, I don’t know). Those who answered the question correctly were given 1 point and who answered no or I don’t know were given 0 points. In this study, a cut-off value of 70% was used to categorize participants’ scores of knowledge into a binary category (high knowledge and low knowledge). The questionnaire and the correct answers and the percentages for each question are displayed in the S1 Appendix.

### Ethical considerations

The Institutional Review Board (IRB) of the University of Jordan, Amman, Hashemite Kingdom of Jordan, reviewed and approved the study protocol in meeting No 2021/18 (reference number: 10/ 2021/ 27975). It was written in accordance with the Helsinki Declaration’s ideals. All participants were asked for their informed consent prior to the introduction of the questionnaire. Once the data was acquired, it was kept in absolute confidence and stored away, with only the lead investigator having access to it.

### Statistical analysis

Data were entered into Microsoft Excel (2016) and then imported into IBM SPSS version 25 (IBM Corp., Armonk, N.Y., USA) for analysis. For each quantitative and categorical variable, descriptive statistics were computed and expressed as either frequency and percentage or mean and standard deviation (SD). Associations between demographic variables and knowledge (binary level) were analyzed using the Chi-square test. Significantly associated variables were included in multivariate regression analysis controlled for potential confounders to assess the independent effect of each variable. Mann-Whitney U-test was used to evaluate the association between the source of information variable and the mean scores of the knowledge score items. A p-value of α < 0.05 and a Confidence Interval of 95% were set to determine the statistical significance of the reported results.

## Results

### Sociodemographic characteristics of the participants

A total of 933 participants met the inclusion criteria and completed the questionnaire. Their sociodemographic characteristics are demonstrated in Table 1. A number of 588 (63%) had a high educational levels (had a diploma degree or higher). In addition, 119 (12.5%) of the participants work in the medical field. Moreover, 165 (17.7%) of participants have a self-reported history of *H*.*pylori* infection and 301 (32.3%) of participants had a family history of *H*. *pylori*. Furthermore, 275 (29.5%) of the participants acquired information about *H*. *pylori* infection from medical sources.

**Table 1:** Sociodemographic characteristics of *H*. *pylori* questionnaire participants (n = 933).

Characteristics	(n/mean)	(%/SD)
**Age**	40.4	±16.1
**Gender**		
Male	493	52.8
Female	440	47.2
**Education Level**		
Low	345	37
High^1^	588	63
**Marital Status**		
Married	602	64.5
Unmarried	331	35.5
**Job Field**		
Medical	119	12.5
Non-medical	487	52.2
No work	327	35
**Type of Residence**		
City	824	88.3
Village	109	11.7
**Self-reported History of *H*. *pylori* Infection**		
Yes	165	17.7
No	768	82.3
**Family History of *H*. *pylori***		
Yes	301	32.3
No	632	67.7
**Source of Information**		
Medical	275	29.5
Non- medical	658	70.5

SD: Standard deviation

^1^ High education: if the participant had a diploma or any higher degrees.

### Knowledge of *H*. *pylori* and its associated factor

The summary of the participant’s correct answers is shown in S1 Table. Out of the 933 participants, 292 (31.3%) and 641 (68.7%) had high and low knowledge levels, respectively. Regarding the nature of *H*. *pylori*, only (37.3%) stated that *H*. *pylori* belong to bacterial species. (76.7%) of the participants chose the stomach as the organ which *H*. *pylori* colonize. The participants were asked about the main route of *H*. *pylori* transmission, and the percentage of correct answers were as the following: food (68.6%), water (63.8%), and saliva (38.4%). For the signs and symptoms of *H*. *pylori* infection, the percentage of the correct symptoms were abdominal pain (75.5%), nausea/vomiting (68.4%), and abdominal bloating (59.8%). Regarding the treatment, (72.7%) of the responses were triple therapy as the main way to eradicate *H*. *pylori* infection. When the participants were asked about the diagnosis of *H*. *pylori*, the responses for the correct answers were as follows: stool antigen (62.2%), endoscopy (62.4%), and urea breath test (13.8%). Regarding general knowledge questions, (44.1%) of the participants acknowledged that more than half of the Jordanian population might be infected with *H*. *pylori*. (69.1%) and (55.2%) of the participants claimed that *H*. *pylori* can be attributed to the development of gastric and duodenal ulcers, respectively. As well as (59.5%) of the participants confirmed that *H*. *pylori* infection can be a life-threatening condition if left untreated. (60.6%) and (63.2%) of the participants asserted that spicy food and chronic stress may worsen the symptoms of *H*. *pylori* infection, respectively. When participants were asked about the long-term risk of developing malignancy from chronic *H*. *pylori* infection, (45.7%) and (26.6%) of the participants responded that *H*. *pylori* infection can lead to gastric cancer and mucosa-associated lymphoid tissue (MALT) B-cell lymphomas, respectively.

Some sociodemographic variables were statistically significantly associated with knowledge as shown in Table 2. Multivariate logistic regression analysis showed significant association with the following variables: job field (Medical: OR = 2.936; 95% CI = 1.718–5.018, p = 0.000; ref: No-work), educational level (Higher education: OR = 1.948; 95% CI = 1.355–2.801, p = 0.000; ref: Lower educational level), self-reported history of *H*. *pylori* (Yes: OR = 2.119; 95% CI = 1.392–3.225, p = 0.000; ref: No), family history of *H*. *pylori* (Yes: OR = 2.129; 95% CI = 1.545–2.935, p = 0.000; ref: No), source of knowledge (medical source: OR = 2.224; 95% CI: 1.535–3.221, p-value: 0.000; ref: non-medical source). Neither age, gender, marital status, nor residency was statistically associated with the knowledge regarding *H*. *pylori* according to the multivariate logistic regression analysis, as reported in Table 3.

**Table 2:** Association between sociodemographic characteristics of the general population and knowledge about *H*. *pylori* (n = 933).

Variables	Level of Knowledge		
	High (n = 292)	Low (n = 641)	P-value
**Age** (mean/SD)	38.5 (±15)	41.2 (±16.5)	0.014
**Gender**:			0.010
Female	156	284	
Male	136	357	
**Marital Status**:			0.092
Married	177	425	
Unmarried	115	216	
**Education Level**:			0.000
Low	74	271	
High	218	370	
**Job Field**:			0.000
Medical	76	43	
Non-medical	125	362	
No-work	91	236	
**Residence**:			0.494
City	261	559	
Village	31	78	
**Self-reported History of *H*. *pylori* infection**			0.000
Yes	83	82	
No	209	559	
**Family History of *H*. *pylori***			0.000
Yes	123	178	
No	169	463	
**Source of Information**:			0.000
Medical	148	127	
Non- medical	144	514	

SD: Standard deviation

**Table 3:** Multivariate logistic regression associations between knowledge and the sociodemographic characteristics of the general population in Jordan.

Covariates	Knowledge		
	OR	CI	p-value
**Age** 38.5(±15)	0.996	0.986–1.006	0.349
**Gender**			
Male	Reference		
Female	1.348	0.998–1.918	0.051
**Education Level**:			
Lower education	Reference		
Higher education	1.948	1.355–2.801	0.000
**Job Field**:			
Medical	2.936	1.718–5.018	0.000
Non-medical	0.852	0.554–1.161	0.411
No-work	Reference		
**Self-reported History of *H*. *pylori* Infection**			
Yes	2.119	1.392–3.225	0.000
No	Reference		
**Family History of *H*. *pylori***			
Yes	2.129	1.545–2.935	0.000
No	Reference		
**Source of Information**:			
Medical	2.224	1.535–3.221	0.000
Non- medical	Reference		

OR: Odds ratio

CI: Confidence interval

### The sources of information and the level of knowledge acquired

As shown in Table 1, the participants in the study utilized various sources of information to attain their knowledge about *H*. *pylori* infection. The majority of the participant 658 (70.5%) used non-medical sources (e.g., family, friends, social media, and TV) to obtain the information. In contrast, only a third 275 (29.5%) of the participant got their information about *H*. *pylori* infection from medical sources such as from a doctor or during their study. By dividing the source of information about *H*. *pylori* infection into medical and non-medical sources; a significant statistical association between the source of information and the level of knowledge has been found (p-value = .000). The Mann-Whitney U test showed that the mean rank of all knowledge items in medical source category significantly exceeds those of the non-medical source group category (p-values < 0.05). The mean ranks and p-values of each question asked are shown in Table 4.

**Table 4:** Mann-Whitney test analysis of medical vs non-medical sources of information regarding the questioned items.

Scale	Medical (n = 292)	Non-medical (641)	P-value
	Mean Rank	Mean Rank	
**Organ Affected Item**	602.44	410.4	.000
**Mode of Transmission Item**	586.52	417.05	.000
**Symptoms Item**	606.34	408.76	.000
**Treatment Item**	570.65	423.68	.000
**Diagnosis Item**	609.59	407.41	.000
**General Knowledge Item**	606.29	408.79	.000

## Discussion

Few studies have been conducted to examine the general population’s awareness regarding *H*. *pylori* infection. Investigating public awareness of *H*. *pylori* infection might aid in determining various methods expected to eradicate the bacteria as well as prevent the long-term consequences of chronic *H*. *pylori* infection. To the best of our knowledge, this study is the first in Jordan to report the level of knowledge about *H*. *pylori* infection and its predictors.

This study included over 900 respondents from outpatient visitors of Jordan University Hospital. In general, the Jordanian population seems to have diminished awareness of *H*. *pylori* infection as the responses showed low awareness in the following items: the nature of *H*. *pylori*, organ colonized by *H*. *pylori*, *H*. *pylori* infection signs and symptoms that are frequent, the definitive treatment and diagnosis and general knowledge about *H*. *pylori* prevalence in Jordan. As well as long-term *H*. *pylori* sequelae include the development of stomach cancer and mucosa-associated lymphoid tissue (MALT) B-cell lymphomas.

Around half of the participants were aware that persistent *H*. *pylori* infection may lead to gastric and duodenal ulcers and gastric cancer. This study showed better awareness compared to previous studies conducted in China, and the United Arab Emirates [15–18] and is quite similar to a research done in Korea [19]. Some sociodemographic characteristics were shown to be connected with greater levels of knowledge in this study. Females have much more information about *H*. *pylori* infection than males. This is in line with previous studies [15–17] and might be linked to the fact that women are often having the role of the family caregiver, and so have more interactions with clinicians to learn about various diseases. Higher education levels and working in the medical area, as predicted, have higher awareness about *H*. *pylori* infection which has been documented in prior studies [15, 17]. In our study, we found respondents who had history of *H*. *pylori* infection or had family members diagnosed with *H*. *pylori* before, tend to have higher knowledge. This finding was supported by the fact that upon diagnosing, more information is provided by the physician about *H*. *pylori*. So, obtaining information directly from a medical source such as a healthcare provider would allow for open-ended and interactive conversation between the healthcare provider and the patient and/or their companion, thus, more understanding of the condition. In contrast, using indirect non-medical sources (media/TV/ radio) lacks such an interaction, leading to ambiguity, false information, and failure to satisfy the patient’s curiosity.

The current study found unsatisfactory outcomes in terms of awareness. This is consistent with comparable research conducted in China, the United Arab Emirates and South Korea [15–17, 19]. Misconceptions in critical areas were discovered. For example, more than half of the participants were not believing in any association between *H*. *pylori* infection and gastric cancer or Mucosa-associated lymphoid tissue (MALT) B-cell lymphomas. This stresses the importance of intervening to correct those misconceptions and to advocate more about how serious *H pylori* infection is. Moreover, we believe that health education can aid in the prevention of *H*. *pylori* infection as this will reduce its impact on the healthcare system and population. Healthcare providers should pay special attention to such dilemmas and implement curricular changes with an emphasis on preventive education. As shown in this study, the non-medical sources are the dominating sources used by the participants. Hence, we put forward and suggest intensive, close surveillance issued by the Jordanian Ministry of Health on social media platforms, TV, and radios to limit and control false and poorly evident health-related information. Thus, it will improve and enhance the knowledge of the population on gaining and obtaining information from authenticated and trusted sources.

### Strengths and limitations

To the best of our knowledge, this is the first survey that has evaluated the degree of knowledge among Jordanians. One of the study’s merits is the face-to-face manner performance, which allowed us to obtain a high response rate and reduce participation bias. This is the first research to look specifically at *H*. *pylori* awareness. The convenience sample approach utilized, and single center involved, may have influenced the generalizability of the results. Self-reported data such as history of *H*. *pylori* infection among participants and their families makes the finding vulnerable to recall bias. Recall bias shows up in many fields and the issue of self-reporting bias represents a key problem and a limitation to the study. However, it is a common approach for gathering data in epidemiologic and medical research. These data are readily available as their collection takes less time and is more cost-effective [20]. Also, self-reported health and disease status are useful indicators of true underlying health status [21, 22]. Despite these limitations, this study was conducted using an interview-based approach which enabled the researchers to instruct participants to report previous infections only if they were certain that they, or their family members, were diagnosed by a medical professional, in addition, anonymity was emphasized to the participants so that they would not under-report in fear of stigma or over-report to seek attention. Finally, optimizing the questionnaire by gastroenterologists and microbiologists helped in correctly communicating the questions to participants.

The lack of a validated questionnaire is one of the barriers we encountered. The authors created this survey since there was no validated questionnaire available to test knowledge of *H*. *pylori* infection. Hence, a validated questionnaire must be developed in the future. Since this is a cross-sectional study, it is difficult to establish a cause-and-effect link. Furthermore, only quantitative measures were utilized to assess awareness. Indeed, sociocultural aspects such as beliefs and tradition were not investigated in our study; these factors should be investigated further in future studies.

## Conclusion

In conclusion, people in Jordan have low awareness regarding *H*. *pylori* infection. Having higher education degree, working in the medical field, history of *H*. *pylori*, family history of *H*. *pylori* and acquiring information from medical sources correlated with a high awareness of *H*. *pylori* infection. Interventions should be implanted to correct the flaws and misconceptions noted in the study to improve general awareness. Developing social programs and campaigns to raise awareness among the general population is a public health imperative in Jordan. Knowing the general principles of *H*. *pylori* infection will reduce the prevalence of *H*. *pylori* infection. In addition, encouraging the population to get information about *H*. *pylori* infection from trusted sources will enhance the understanding of the general population about routes of transmission and thus diminishes the consequences of *H*. *pylori* infection, including the occurrences of gastric cancer secondary to such infection. Future studies should implement a validated questionnaire, consider multicenter cross-sectional study, and measure the prevalence of *H*. *pylori* among the target population.

## Supporting information

S1 Appendix Questionnaire (Arabic and English versions). This is the Arabic and English versions of the questionnaire administered to participants.
(DOCX)

S1 Table Correct answers table. This is the participants’ percentage to each correct answer.
(DOCX)
