# Supplementary material for: Knowledge and information sources towards Helicobacter pylori in Jordan
Source: PLoS One. 2023 Mar 8;18(3):e0278078. doi: 10.1371/journal.pone.0278078 (PMC9994704; doi:10.1371/journal.pone.0278078)
Supplement: S1 Appendix — This is the Arabic and English versions of the questionnaire administered to participants. (DOCX) [file pone.0278078.s001.docx]

**النسخة العربية**

**Arabic Version**

**معلومات أساسية :**

1 - العمر ............

2 - الجنس: * ذكر

* انثى

3 - الحالة الاجتماعية *متزوج

*غير متزوج ( أعزب, أرمل )

4 - المستوى التعليمي

*مستوى عالي ( دبلوم أو أعلى )
 * مستوى منخفض (ثانوي او أقل)

5 - المجال الوظيفي *طبي

*غير طبي

* لا أعمل

6- هل سبق لك الإصابة بالجرثومة الحلزونية:
 * نعم
 * لا

7- هل سبق لأحد من عائلتك الإصابة بالجرثومة الحلزونية:
 * نعم
 * لا

8- من أين حصلت على المعلومات الخاصة بالجرثومة الحلزونية:
 * مصدر طبي ( عن طريق ممارس صحي او من حصلت عليه خلال الدراسة)
 *مصدر غير طبي ( عن طريق الأهل/الأصدقاء أو التلفاز/ الراديو/ منصات التواصل الاجتماعي)

9- مكان الاقامة الحالي:

*الريف

*المدينة

**المعرفة:**

1 – هل طبيعة الجرثومة الحلزونية كالأتي

*فيروس 1- نعم 2- لا 3- لا أعلم

*بكتيريا 1- نعم 2- لا 3- لا أعلم

*فطريات 1- نعم 2- لا 3- لا أعلم

*طفيليات 1- نعم 2- لا 3- لا أعلم

2 – هل الآتي العضو الآتي تستوطنه وتعيش فيه الجرثومة الحلزونية

*الدماغ 1- نعم 2- لا 3- لا أعلم

*القلب 1- نعم 2- لا 3- لا أعلم

*المعدة 1- نعم 2- لا 3- لا أعلم

*الكبد 1- نعم 2- لا 3- لا أعلم

*الرئة 1- نعم 2- لا 3- لا أعلم

3 – هل الطرق الآتية التي تتم عن طريقها انتشار عدوى الجرثومة الحلوزنية:

*الدم 1- نعم 2- لا 3- لا أعلم

*المياه الملوثة 1- نعم 2- لا 3- لا أعلم

*الطعام الملوث 1- نعم 2- لا 3- لا أعلم

*الهواء 1- نعم 2- لا 3- لا أعلم

*الاتصال الجنسي 1- نعم 2- لا 3- لا أعلم

*الاسطح الملوثة 1- نعم 2- لا 3- لا أعلم

4 –الاعراض الآتية ممكن أن تحصل أثناء الإصابة بالجرثومة الحلزونية:
 *صداع 1- نعم 2- لا 3- لا أعلم

*ألم في المعدة 1- نعم 2- لا 3- لا أعلم

*الم في الظهر 1- نعم 2- لا 3- لا أعلم

*الم في الصدر 1- نعم 2- لا 3- لا أعلم

*غثيان او استفراغ 1- نعم 2- لا 3- لا أعلم

*كحة 1- نعم 2- لا 3- لا أعلم

*انتفاخ او تجشؤ 1- نعم 2- لا 3- لا أعلم

*الم و احمرار في الجلد 1- نعم 2- لا 3- لا أعلم

*ضيق في النفس 1- نعم 2- لا 3- لا أعلم

*حرارة 1- نعم 2- لا 3- لا أعلم

*تعب 1- نعم 2- لا 3- لا أعلم

5 – هل يتم تشخيص الجرثومة الحلزونية:

*الفحص السريري 1- نعم 2- لا 3- لا أعلم

*فحص تحليل البراز 1- نعم 2- لا 3- لا أعلم

*فحص الدم 1- نعم 2- لا 3- لا أعلم

*فحص تحليل البول 1- نعم 2- لا 3- لا أعلم

*المنظار و الخزعة 1- نعم 2- لا 3- لا أعلم

*مادة اليوريا في النفس 1- نعم 2- لا 3- لا أعلم

6 – كيف يمكن علاج الجرثومة الحلزونية

*علاجات غير دوائية 1- نعم 2- لا 3- لا أعلم

*علاجات دوائية 1- نعم 2- لا 3- لا أعلم

*تعالج ذاتياً 1- نعم 2- لا 3- لا أعلم

7 – ماذا تعتقد بالاتي بالنسبة للجرثومة الحلزونية:

*على الاقل 50% من سكان الاردن لديهم عدوى الجرثومة الحلزونية.

1 - نعم 2- لا 3- لا أعلم

*الجرثومة الحلزونية قد تهدد حياة الانسان اذا اصبحت شديدة و لم تعالج

1- نعم 2- لا 3- لا أعلم

*التوتر المزمن قد يزيد من أعراض الجرثومة الحلزونية

1- نعم 2- لا 3- لا أعلم

*الاطعمة الحارة قد تزيد من حدة أعراض الجرثومة الحلزونية

1- نعم 2- لا 3- لا أعلم

*الجرثومة الحلزونية يمكن ان تسبب قرحة في المعدة
 1- نعم 2- لا 3- لا أعلم

*الجرثومة الحلزونية يمكن ان تسبب قرحة في الاثني عشر
 1- نعم 2- لا 3- لا أعلم

*الجرثومة الحلزونية يمكن ان تسبب سرطان المعدة
 1- نعم 2- لا 3- لا أعلم

*الجرثومة الحلزونية يمكن ان تسبب سرطان الأنسجة اللمفاوية؟

1- نعم 2- لا 3- لا أعلم

**English version**

**Sociodemographic :**

1 - Age............

2 - Gender: * Male

* Female

3 - Marital status *Married

*Unmarried (single, widowed)

4 - Educational level

* High level (diploma or higher)
 * Low level (secondary or less)

5 – Work field *Medical

*Non-medical

* I don't work

6- Have you ever had Helicobacter pylori infection:

*Yes
 * No

7- Has anyone in your family ever had Helicobacter pylori infection:

*Yes

*No

8- Where did you get the information about Helicobacter pylori:

*Medical sources (through health practitioner or during the study))
* Non-medical sources (through family/friends or TV/radio/social media platforms)

9- Current place of residence :

*Village

*City

**Knowledge:**

1 – what Is the nature of Helicobacter pylori:

*Virus 1- Yes 2- No 3- I don't know

*Bacterial 1- Yes 2- No 3- I don't know

*Fungi 1- Yes 2- No 3- I don't know

*Parasites 1- Yes 2- No 3- I don't know

2 – which of the following organs does Helicobacter pylori colonies in ?

*Brain 1- Yes 2- No 3- I don't know

*Heart 1- Yes 2- No 3- I don't know

*Stomach 1- Yes 2- No 3- I don't know

*Liver 1- Yes 2- No 3- I don't know

*Lung 1- Yes 2- No 3- I don't know

3 – what is the transmission route of Helicobacter pylori?

*Blood 1- Yes 2- No 3- I don't know

*Contaminated water 1- Yes 2- No 3- I don't know

*Contaminated food 1- Yes 2- No 3- I don't know

*Air 1- Yes 2- No 3- I don't know

*Sexual contact 1- Yes 2- No 3- I don't know *Contaminated surfaces 1- Yes 2- No 3- I don't know

4 – The following symptoms can occur during infection by Helicobacter pylori:

* Headache 1- Yes 2- No 3- I do not know

*Stomach pain 1- Yes 2- No 3- I don't know

*Back pain 1-Yes 2- No 3- I don't know

*Chest pain 1-Yes 2- No 3- I don't know

*Nausea or vomiting 1- Yes 2- No 3- I don't know

*Cough 1- Yes 2- No 3- I don't know

*Abdominal bloating 1- Yes 2- No 3- I don't know

* Rash in the skin 1- Yes 2- No 3- I don't know

*Shortness of breath 1- Yes 2- No 3- I don't know

*Fever 1- Yes 2- No 3- I don't know

*Fatigue 1- Yes 2- No 3- I don't know

5 – Helicobacter Pylori diagnosed by :

*Clinical examination 1- Yes 2- No 3- I do not know

*Stool analysis test 1- Yes 2- No 3- I do not know

*Blood test 1- Yes 2- No 3- I don't know

*Urine analysis test 1- Yes 2- No 3- I don't know

*Endoscopy & biopsy 1- Yes 2- No 3- I do not know

*Urea breath test 1- Yes 2- No 3- I don't know

6 – How to treat Helicobacter pylori

*Herbal treatments 1- Yes 2- No 3- I don't know

*Medical treatments 1-Yes 2- No 3- I don't know

*Self-limited disease 1- Yes 2- No 3- I don't know

7 – What do you think about Heliobacterium pylori:

At least 50% of Jordan's population has Helicobacter pylori infection:
1- Yes 2- No 3- I don't know.

Helicobacter pylori may threaten human life if it becomes severe and not treated:
1- Yes 2- No 3- I don't know.

Chronic stress may increase symptoms of caused by helicobacter pylori infection:

1- Yes 2- No 3- I don't know.

Spicy foods may worsen the symptoms of Helicobacter pylori infection:

1- Yes 2- No 3- I don't know.

Helicobacter pylori can cause stomach ulcers:
1- Yes 2- No 3- I don't know.

Helicobacter pylori can cause duodenal ulcers:
1- Yes 2- No 3- I don't know.

Helicobacter pylori can cause stomach cancer:
1- Yes 2- No 3- I don't know.

Helicobacter pylori can cause mucosa-associated lymphoid tissue (MALT)?

1- Yes 2- No 3- I don't know
