# Supplementary material for: Knowledge and information sources towards Helicobacter pylori in Jordan
Source: PLoS One. 2023 Mar 8;18(3):e0278078. doi: 10.1371/journal.pone.0278078 (PMC9994704; doi:10.1371/journal.pone.0278078)
Supplement: S1 Table — This is the participants’ percentage to each correct answer. (DOCX) [file pone.0278078.s002.docx]

**Participants’ knowledge about *Helicobacter pylori***

| **Item** | | **Correct answer** | **Correct answer, n (%)** |
| --- | --- | --- | --- |
| **Nature of *H. pylori*** | | Bacteria | 348 (37.3%) |
| **Organs affected by *H. pylori*** | |  |  |
| Stomach | | Yes | 715 (76.6%) |
| Brain | | No | 450 (48.2%) |
| Heart | | No | 426(45.7%) |
| Liver | | No | 332(35.6%) |
| Lung | | No | 391(41.9%) |
| Kidney | | No | 362(38.8) |
| **Route of transmission of *H. pylori*** | |  |  |
| Food | | Yes | 640 (68.6%) |
| Water | | Yes | 595 (63.8%) |
| Saliva | | Yes | 358 (38.4%) |
| Blood | | No | 370(39.7%) |
| Air | | No | 505(54.1%) |
| Sexual contact | | No | 455(48.8%) |
| Surface | | No | 313(33.5%) |
| ***H. pylori* symptoms** | |  |  |
| Abdominal pain | | Yes | 704 (75.5%) |
| Nausea and/or vomiting | | Yes | 638 (68.4%) |
| Abdominal bloating | | Yes | 558 (59.8%) |
| Headache | | No | 243(26%) |
| Chest pain | | No | 324(34.7%) |
| Backache | | No | 358(38.4%) |
| Nausea | | No | 638(68.4%) |
| Cough | | No | 429(46%) |
| Dyspnea | | No | 358(38.4%) |
| Rach | | No | 440(47.2%) |
| Fever | | No | 223(23.9%) |
| Fatigue | | No | 74(7.9%) |
| ***H. pylori* treatment** | |  |  |
| Triple therapy | | Yes | 678(72.7%) |
| Herbal therapy | | No | 402(43.1%) |
| Self-limited | | No | 640(68.6%) |
| ***H. pylori* diagnosis** | |  |  |
| Stool antigen | | Yes | 622 (66.7%) |
| Urea breath test | | Yes | 129 (13.8 %) |
| Endoscopy | | Yes | 582 (62.4%) |
| Clinical diagnosis | | No | 453(48.6%) |
| Urine analysis | | No | 380(40.7%) |
| Blood test | | No | 188(20.2%) |
| **General knowledge** | |  | |
| More than 50% of the Jordanian population have *H. pylori* | | Yes | 411 (44.1 %) |
| *H. pylori* is considered as life-threatening condition if left untreated | | Yes | 555 (59.5 %) |
| Things that may worsen *H. pylori* symptoms | Chronic Stresses | Yes | 590 (63.2 %) |
|  | Spicy food | Yes | 565 (60.6 %) |
| Possible Consequences of *H. pylori* infection | Gastric ulcer | Yes | 645 (69.1 %) |
|  | Duodenal ulcer | Yes | 515 (55.2 %) |
|  | Gastric cancer | Yes | 426 (45.7%) |
|  | Mucosa-associated lymphoid tissue | Yes | 204 (26.6 %) |

- The datasets gathered and analyzed during the current study are available at: [10.6084/m9.figshare.21991844](https://doi.org/10.6084/m9.figshare.21991844).
